# Road-Network-Map-Assisted Vehicle Positioning Based on Pose Graph Optimization

**DOI:** 10.3390/s23177581

**Published:** 2023-08-31

**Authors:** Shuchen Xu, Yongrong Sun, Kedong Zhao, Xiyu Fu, Shuaishuai Wang

**Affiliations:** National Key Laboratory of Helicopter Aeromechanics, College of Automation Engineering, Nanjing University of Aeronautics & Astronautics, Nanjing 211106, China

**Keywords:** visual odometry, road network map, map correction points, optimization and prediction model, pose graph optimization

## Abstract

Satellite signals are easily lost in urban areas, which causes difficulty in vehicles being located with high precision. Visual odometry has been increasingly applied in navigation systems to solve this problem. However, visual odometry relies on dead-reckoning technology, where a slight positioning error can accumulate over time, resulting in a catastrophic positioning error. Thus, this paper proposes a road-network-map-assisted vehicle positioning method based on the theory of pose graph optimization. This method takes the dead-reckoning result of visual odometry as the input and introduces constraints from the point-line form road network map to suppress the accumulated error and improve vehicle positioning accuracy. We design an optimization and prediction model, and the original trajectory of visual odometry is optimized to obtain the corrected trajectory by introducing constraints from map correction points. The vehicle positioning result at the next moment is predicted based on the latest output of the visual odometry and corrected trajectory. The experiments carried out on the KITTI and campus datasets demonstrate the superiority of the proposed method, which can provide stable and accurate vehicle position estimation in real-time, and has higher positioning accuracy than similar map-assisted methods.

## 1. Introduction

The global satellite positioning system utilizes satellites in Earth’s orbit to provide positioning information [1], which is widely used in vehicle navigation systems, relying on its high cost-effectiveness [2]. However, due to the inherent shortcomings of satellite positioning principles, the global satellite positioning system has problems, such as unstable signals and low local positioning accuracy in urban canyon environments [3,4]. Inertial sensor [5,6,7] or wheel odometry [8,9,10] data are usually introduced into the positioning system to provide accurate and robust vehicle positioning information. But the external parameters between different sensors in the multi-sensor fusion system are easily affected by mechanical deformation and harsh environments, which will lead to a reduction in system positioning accuracy. At the same time, the high price of inertial devices and the inevitable slipping problem of wheel odometry limit their application scenarios.

With the development of computer vision technology, different kinds of machine vision systems are introduced into navigation and positioning devices [11]. A visual sensor has the advantages of convenient use, light weight, and high cost-effectiveness [12], so many researchers have used it to solve vehicle positioning problems in recent years. There are two mainstream visual-based vehicle positioning methods: one type utilizes the pre-built geographic marker image library. The best match of the current image collected by the car-mounted camera is searched for in the image database to locate the vehicle [13,14,15]. This method has good positioning performance, but requires professional personnel to collect environmental information in advance and build the image database, which is time-consuming and labor-intensive. The high cost of updating and maintenance in the later stage is also an unbearable issue. Another type of method tracks the motion status of the vehicle by inputting a series of images into the Visual Odometry (VO), which relies on the dead-reckoning principle [16,17,18,19]. As driving time and distance increase, errors gradually accumulate, which leads to a decrease in positioning accuracy.

To solve the cumulative drift problem of VO, some scholars have adopted methods based on Visual Simultaneous Localization and Mapping (VSLAM) [20,21,22,23,24]. However, VSLAM requires the vehicle to repeatedly drive through historical scenes and construct a loop closure to reduce positioning error, which affects vehicle travel planning. Some researchers have fused information collected by the camera and other sensors to suppress drift error [25,26,27,28,29]. However, introducing additional sensors will increase the hardware cost and data processing complexity of the navigation system. On the other hand, the cost of obtaining map information is decreasing continually with the rapid development of the Internet [30,31,32,33]. Combining constraints from the map can effectively suppress VO positioning drift. This paper proposed a vehicle positioning method based on the theory of pose graph optimization, which introduces constraints from a point-line form road network map to suppress the original positioning error of VO, without needing the loop closure trajectory or other sensors’ assistance. We first propose a road network model based on the Road Network Basic Element (RNBE), which simplifies the complex original map information and also efficiently uses the map information to assist in vehicle positioning. Then, we design the joint judgment criteria of length/angle based on RNBE and use the road network map data and real-time vehicle positioning prediction results to select map correction points. The Road Network Assisted Position (RNAP) algorithm is designed to optimize the original trajectory by comprehensively taking into account the short-term accuracy of the output of VO and the global consistency of the map correction points. Based on the latest VO calculation result and optimized trajectory, stable and accurate vehicle position estimation can be predicted in real-time. The experiments carried out on the KITTI and campus datasets demonstrate that the method proposed in this paper can effectively overcome the problem of cumulative drift error in VO and has higher positioning accuracy compared to similar algorithms.

The organizational structure of the remaining parts of this paper is as follows. Section 2 reviews related works of map-assisted positioning methods. Section 3 provides an overall overview of this system. The RNAP algorithm is described in detail in Section 4. Section 5 presents the experimental results. Conclusions are given in Section 6.

## 2. Related Work

More and more vehicle positioning systems have been using VO to assist in positioning in recent years due to its continuously improving positioning performance and extremely advantageous cost-effectiveness. Similar to the principle of traditional odometry, VO achieves positioning by calculating the incremental position and attitude between adjacent frames, which possesses short-term accuracy. The error in matching will gradually accumulate over time, making VO unsuitable for remote navigation. The conventional methods detect the loop closure and perform global BA to optimize the position and attitude of the vehicle and suppress drift error [34,35]. But the actual driving trajectory may not contain loop closures, and the corrected positioning results do not have real-time performance, making this unsuitable for applications in vehicle driving. Integrating other sensors into the positioning system can suppress positioning errors in real-time [36,37], but will increase the overall cost.

Compared with the above methods, introducing constraints from maps to suppress the VO drift error is effective and economical. Maps contain different types of environmental information, which introduce constraints from multiple perspectives. Ref. [38] proposes a vehicle localization method using a monocular camera and compact semantic map. Xiao stores landmark information in a custom map and identifies landmarks in monocular images through a neural network to extract geometric features. The vehicle position is optimized by minimizing customized reprojection errors. Ref. [39] pays special attention to the three-dimensional surface of buildings around the vehicle, generating the segmented point cloud from stereo images and matching it with the reference point cloud of OpenStreetMap. The designed RLE framework can achieve lane-level positioning, but its applicability is not strong in rural areas with sparse buildings. Ref. [40] combines sequential Monte Carlo tracking with the map–image matching relationship. Zhou encodes semantic information in images and maps to assist with positioning, which can effectively address challenges in different environments. Ref. [41] utilizes the mature SeqSLAM algorithm for location recognition. The position information identified is used as the observation value of the filter and fused with the VO positioning information for long-term navigation.

Some scholars have also proposed positioning methods that combine VO output with the map information through the probability model, which makes the positioning result more robust. Ref. [42] divides the original trajectory of VO into multiple straight segments and aligns the trajectory segments with the 2D map to correct drift error. This Snap method assumes that the map is composed of several straight lines, but roads may be curved in the real world, which restricts the application scenarios. Ref. [43] utilizes V2V communication technology for multi-vehicle collaborative positioning. When two vehicles converge, a merged query sequence is formed and matched with the map to achieve precise positioning. Refs. [44,45] extract the sequence of heading-length values from the trajectory of the proprioceptive sensor odometry. Cheng achieves accurate and robust vehicle positioning results by matching the heading sequence with a preprocessed heading length map. Based on the trajectory fast chamfer matching technology, ref. [46] incorporates map data as additional clues into the observation model of the Monte Carlo positioning framework. The OpenStreetSLAM method can effectively compensate for the drift error generated by VO over time. However, the distortion of the VO trajectory will lead to incorrect matching and reduce positioning accuracy. Ref. [47] proposes the Turning Point Filtering (TPF) algorithm, which does not force the corrected trajectory to fit onto the edges of the map. Under the particle filtering framework, Jin designs a flexible turning point filtering mechanism, which only optimizes the turning points of the trajectory in situations with low uncertainty. Although the TPF algorithm has high computational efficiency, it is not suitable for areas with high-curvature roads. Ref. [48] proposes a curved road representation method based on anchors, which can capture the main curved points of the trajectory. Gu designs a Multi-Position Joint Particle Filtering (MPJPF) framework to correct positioning results, which can avoid catastrophic consequences caused by forced positioning in uncertain situations. However, as the number of joint particles increases, the computational time and cost rapidly increases.

This paper proposes a road-network-map-assisted vehicle positioning method. We initialize particles around the key nodes of the point-line geometric map. When the vehicle position meets the preset joint judgment criteria of length/angle, the node particle with the highest matching degree is selected as the map correction point to optimize the original vehicle trajectory. We predict the vehicle positioning result at the latest moment based on the latest VO calculation result and optimized trajectory. This method can effectively constrain the odometry drift and improve vehicle positioning accuracy with minimal additional cost.

The main contributions of this paper are as follows:

- The constraints are introduced from the point-line form road network map, with no need for richer map information to suppress the VO drift error. The map data required for the proposed method are small, making them suitable for large-scale scenarios;
- The new model of the road network map designed based on RNBE can accurately describe the shape of roads and the topological relationships between different RNBEs. Furthermore, the joint judgment criteria of length/angle based on RNBE provides assistance for selecting map correction points;
- The proposed optimization and prediction model is able to effectively improve the trajectory accuracy through the RNAP algorithm in the optimization stage, and predict vehicle position estimation in real-time by combining the latest VO calculation result and optimized trajectory in the prediction stage;
- The experiments carried out on public and campus datasets demonstrate that this method can effectively improve vehicle positioning accuracy.

## 3. System Overview

This paper assumes that the vehicle suddenly encounters a global positioning system malfunction during driving, resulting in the original onboard navigation system being unable to be located. At this moment, the position and attitude of the vehicle are known. We can turn on the onboard VO to navigate, and the positioning error of VO is suppressed by introducing constraints from the regional road network map.

### 3.1. The Road Network Map Based on RNBE

The regional road network map required in this paper is the point-line form geometric map, whose source is public map websites. Required map data include topological connection relations between different roads and geometric shape information of the single road. The OpenStreetMap website collects data through manual measurement, satellite measurement, and aerial photography, used as an open database for users worldwide. The OSM road network map uses three basic elements, Node, Way, and Relation, to describe the overall road network. The Node represents a single point element of the map, including its latitude and longitude information. The Way is an ordered list composed of a series of Nodes. The Relation represents the connection between Nodes and Ways.

We define the smallest inseparable road section as the Road Network Basic Element (RNBE). As its name implies, RNBE is the basic element of the road network. This paper proposes a road network model that uses road intersections as support points and RNBEs to represent the roads between adjacent support points. Furthermore, we define the support points of the road network as Turning Points (Tp). The first and last nodes of any RNBE are Tps, which contain the topological connection relationships between different RNBEs. For a single RNBE, the shape of the road is approximated by sequentially connecting internal nodes, which are defined as Skeleton Points (Sp).

The structural composition of a typical RNBE is shown in Equation (1), where $Tp_{Start}$ represents the Head-Tp, $Tp_{End}$ represents the Tail-Tp, and $\left\{Sp_{1},Sp_{2},\cdots ,Sp_{n}\right\}$ represent the set of Sps.

(1) $$\left\{Tp_{Start},Sp_{1},Sp_{2},\cdots ,Sp_{n},Tp_{End}\right\}$$

**Figure 1:**
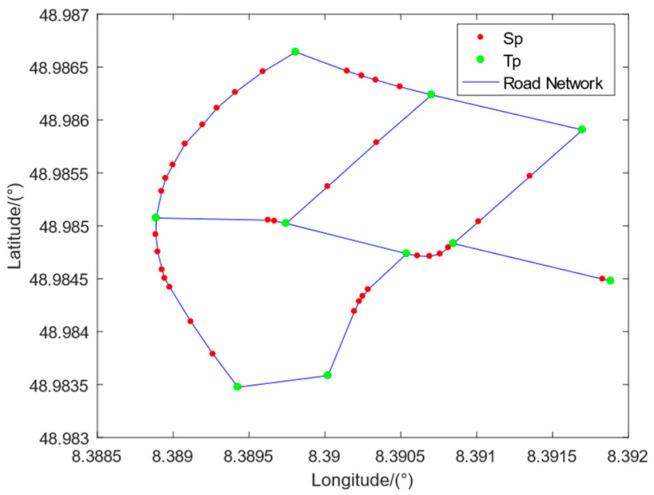
Regional road network map diagram based on RNBE. The green Tps contain the topological connection relationships between different RNBEs, and the red Sps′ connection lines approximate the shape of the RNBE.

The regional map under the road network model designed in this paper is shown in Figure 1. We used twelve independent RNBEs to represent the original road network, in which different RNBEs with the same Tp have connectivity. The internal shape of RNBE is approximated by connecting Sps. There are fewer internal Sps in the near-straight RNBE, while there are more internal Sps in the curved RNBE, ensuring the maximum similarity between the connection of Sps and the shape of the original road.

### 3.2. System Flow

**Figure 2:**
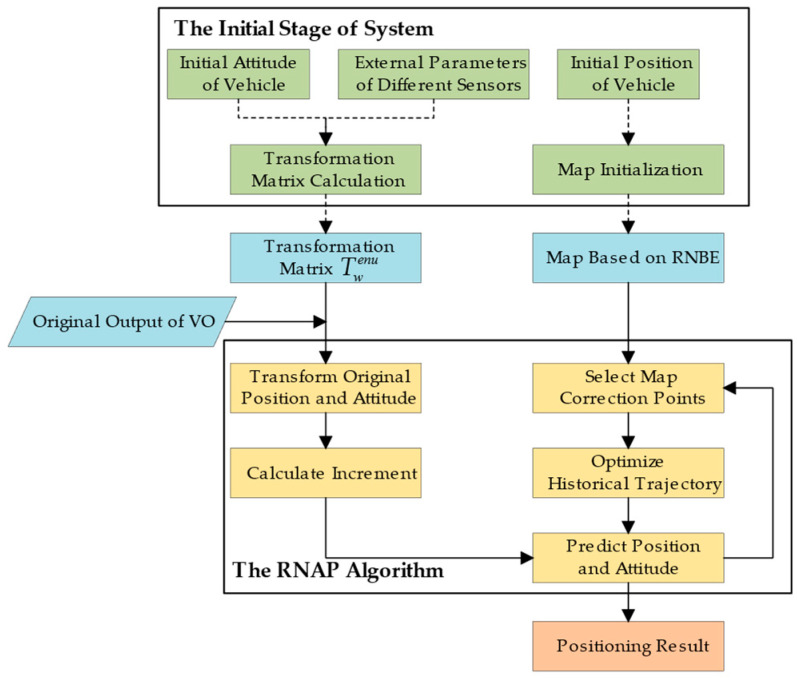
Flowchart of the overall system: green blocks are the work in the initial stage, blue blocks are the input of RNAP algorithm, yellow blocks are the processes carried out in a loop, and the orangeblock is the output.

The flowchart of the positioning system designed in this paper is shown in Figure 2. In the initial stage, we first load the regional road network map based on the initial position of the vehicle and initialize it. The initialization work includes converting the original map into the form of the map based on RNBE and locating the initial road section to which the vehicle belongs. The transformation matrix from the visual world coordinate system to the ENU coordinate system $T_{w}^{enu}$ is calculated based on the initial attitude of the vehicle and external parameters of the sensors, and is an invertible matrix.

The position and attitude of the vehicle output by VO is in the visual world coordinate system. We convert it to the ENU coordinate via $T_{w}^{enu}$ and calculate the increment $\Delta T_{enu}$ at the latest moment. Then, we predict the latest position and attitude of the vehicle by combining $\Delta T_{enu}$ with the optimized historical trajectory (initially the original trajectory). Meanwhile, the map correction points are selected based on the preset joint judgment criteria of length/angle. Visual odometry provides local position and attitude factors, and the map correction points provide global position factors. These two kinds of factors are inputted into the global pose graph to construct the optimization problem, which can correct the historical trajectory of the vehicle. We continuously select map correction points and optimize historical trajectory by repeating the above process. An accurate and stable vehicle position can be predicted in real time by attaching increments calculated by VO to optimized trajectories.

Traditional methods generally derive particles from raw trajectory points and design filtering algorithms to suppress VO drift error. This paper adopts a different approach, which derives particles from the key nodes in RNBEs and selects map correction points through preset judgment criteria. Map correction points are independent of each other, which can not only reduce the impact of wrong corrections but also provide better real-time performance.

## 4. The RNAP Algorithm

The fundamental module for the vehicle positioning system designed in this paper is Visual Odometry. The Road Network Assisted Position (RNAP) algorithm introduces constraints from the road network to suppress the accumulated error of VO and achieve precise positioning. Road network data and the RNAP algorithm play an auxiliary but essential role. The specific flow of RNAP is shown in the black box in Figure 2.

### 4.1. System Initialization

This section introduces the initialization work of the system. Firstly, the regional road network map is loaded based on the vehicle’s initial position and is converted into the map form described in Section 3.1. After that, we locate the initial road segment to which the vehicle belongs in the road network map.

#### 4.1.1. Map Initialization

The raw data of the road network map provided by OpenStreetMap are the set of Ways, where a single Way may pass through multiple road intersections. For the regional road network, we calculate intersections between different Ways and divide them into multiple RNBEs. For a single RNBE, we traverse the Head-Tp and Tail-Tp of the remaining RNBEs in the set. If two Tps are located at the same road intersection, we judge that the RNBEs to which the Tps belong are connected. The connectivity of all RNBEs within the road network can be calculated by traversing the RNBE set and repeating the above processes.

The number of internal Sps in the near-straight RNBE is, relatively, less because its internal road shape is single, for which we can connect a small amount of Sps to approximate the road shape. We increase the number of internal Sps of each RNBE during the initialization phase to provide more data support for trajectory correction. Taking the broken line segment $Tp_{Start}-Sp_{1}$ as the example, if the length Len of $Tp_{Start}-Sp_{1}$ is less than 10, we judge that the distance between two Nodes is small with no need to add Sp. Otherwise, the number of Sps needing to be added is calculated according to Equation (2) and added using the linear interpolation method, where ${\left(\cdot \right)}_{floor}$ represents rounding down.

(2) $$Num={\left(Len/10+0.5\right)}_{floor}$$

We calculate the Head-Orientation and Tail-Orientation of each RNBE after adding Sps. If a certain RNBE has no internal Sps, we use $\vec{Tp_{Start}Tp_{End}}$ to define its Head-Orientation and Tail-Orientation. If the set of Sps is not empty, the Head-Orientation is $\vec{Tp_{Start}Sp_{1}}$ and the Tail-Orientation is $\vec{Sp_{n}Tp_{End}}$. The calculated Head-Orientation and Tail-Orientation will be used to select Tp correction points and replace the RNBE to which the vehicle belongs. We will introduce this content in detail in Section 4.2.2.

#### 4.1.2. Initial Road Section Locating

**Figure 3:**
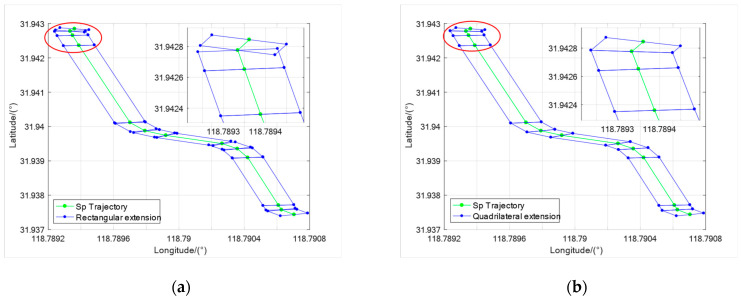
The green dot lines represent the trajectory of Sps: (**a**) represents the rectangular extension of the RNBEs, and from the magnified effect picture of the red circle part, the existence of the Overlapping area and Blank area is obvious; (**b**) represents the quadrilateral extension of the RNBEs. The Overlapping area and Blank area have been eliminated in the red circle part.

To fully utilize the road network information, we calculated the RNBE to which the vehicle belongs based on its location in the initial stage. The point-line form road network map represents the shape of roads through connecting lines between Nodes. The Nodes are usually located in the center of the road, and the connecting lines of the Nodes are the centerlines of the road. Using the internal Sps as support, we design the rectangular expansion of RNBE, with an extension value of 1.5 times the road width, as shown in Figure 3a. The rectangular expansion of RNBE will form a Blank area and an Overlapping area, as shown in the enlarged image of Figure 3a. The existence of the Overlapping-area will increase the difficulty of locating the internal road section in RNBE, and the Blank area will cause the loss of road shape and increase the probability of positioning failure.

To avoid the impact of the Overlapping area and Blank area, we further expand RNBE quadrangularly on the basis of rectangular expansion. As shown in Figure 3b, for the Overlapping area, intersection points of the road boundary are taken as new vertexes. For the Blank area, we use the intersection points of extension lines of the road boundaries as new vertexes and expand RNBE into a quadrilateral. The road section to which the vehicle belongs depends on the inclusion relationship between its initial position and the RNBE quadrilateral expansion. There are multiple possible road sections where the vehicle may belong at the road intersection, so further positioning is needed based on the initial heading of the vehicle. After successful positioning, we adjust the Head-Tp, Tail-Tp, and internal Sps′ order of the RNBE based on the vehicle’s heading.

### 4.2. Map Correction Points

We select map correction points from the Tail-Tp and internal Sps of RNBE. This section first introduces the generic particle derivation method and similarity judgment criteria used for Tp/Sp correction points. Then, we show the method for selecting map correction points in detail.

#### 4.2.1. Particle and Similarity

In the point-line form of the road network map, Tps are usually in the center of the road intersections, and Sps are usually at the centerlines of the road. During actual driving, it is difficult to ensure that the vehicle is always at the centerline of the road due to the uncertainty of the vehicle trajectory. We aimed to derive particles from key Nodes of RNBE to cover all the possible positions of the vehicle. For a single Node, we connected it with particles derived from it. The angle between the connecting line and the positive direction of longitude is a random variable of 0–2π, which can ensure that the derived particles are distributed around the Node evenly. The distance between the Node and its derived particles is a Gaussian distribution with a mean of zero and a standard deviation of one-sixth of the road width. Nodes are usually in the center of the road. Half of the road width is the distance from the Node to the road edge. By utilizing the property of Gaussian distribution, 99.73% of the data is distributed within the interval (μ−3σ,μ+3σ). Choosing one-sixth of the road width as the standard deviation can ensure that the majority of the derived particles are within the road area. The closer the area is to the centerline of the road, the more particles will be derived, which is in line with the daily driving characteristics of the vehicle.

**Figure 4:**
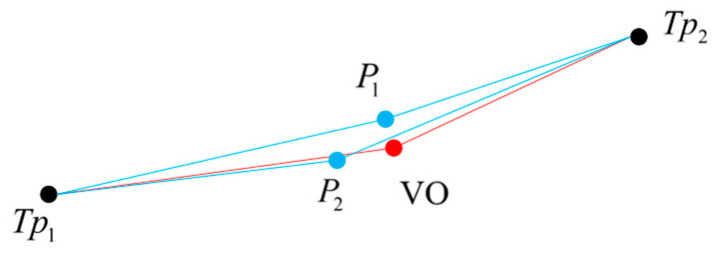
The illustration for calculating the similarity between Nodes: the black points represent the Head-Tp and Tail-Tp of the RNBE, the red point represents the position of the output of VO, and the blue points represent two derived particles of the Sp.

Refs. [45,46] have proposed mature methods to calculate similarity by fusing length/angle conditions. We improve their methods to adapt to the map based on RNBE. As shown in Figure 4, $P_{1}$ and $P_{2}$ are two derived particles of Sp, and $Tp_{1}$ and $Tp_{2}$ are the Head-Tp and Tail-Tp of the RNBE. Considering the orientations of the connecting lines, $P_{2}$ and the output position of VO are more similar. However, considering the lengths of the connection lines, $P_{1}$ and the output position of VO are more similar.

Using $Tp_{1}$ as the reference point, the relative length error between $P_{1}$ and the position of VO is calculated using Equation (3), and the angle error is calculated using Equation (4).

(3) $$Error_{l}(Tp_{1},VO,P_{1})=\frac{\left|Len(Tp_{1},VO)-Len(Tp_{1},P_{1})\right|}{Len(Tp_{1},VO)}$$

(4) $$Error_{A}(Tp_{1},VO,P_{1})=arccos\left(\frac{\vec{Tp_{1}VO}\cdot \vec{Tp_{1}P_{1}}}{\left|\vec{Tp_{1}VO}\right|\cdot \left|\vec{Tp_{1}P_{1}}\right|}\right)$$

The similarity $S_{Tp_{1}}^{P_{1}/VO}$ relative to the reference point $Tp_{1}$ between $P_{1}$ and the position of VO is calculated using Equation (5).

(5) $$S_{Tp_{1}}^{P_{1}/VO}=\alpha \times S_{l}+(1-\alpha )\times S_{A}$$

where $S_{l}=exp\left[-\left(Error_{l}(Tp_{1},VO,P_{1})+Error_{l}(Tp_{1},P_{1},VO)\right)/2\right]$, $S_{A}=1-Error_{A}(Tp_{1},VO,P_{1})/\pi$, and α is the weighting factor.

For the Tail-Tp of RNBE and its derived particles, the Head-Tp is selected as the reference point to calculate similarity. For the internal Sp in RNBE and its derived particles, we calculate similarity by using Head_Tp and Tail_Tp as reference points, respectively, and take the average value as the final similarity.

#### 4.2.2. Map Correction Point Selecting

##### Tp Correction Point Selecting

**Figure 5:**
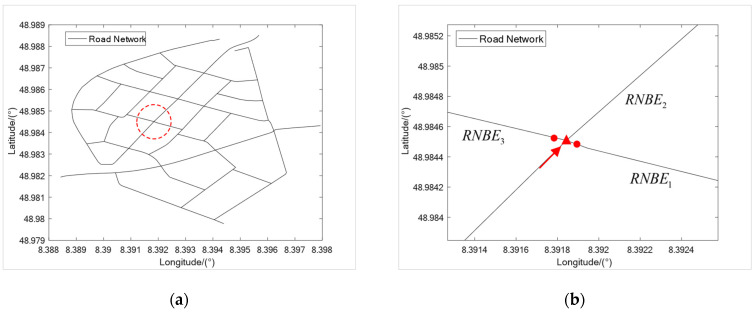
(**a**) Regional road network map. (**b**) Enlarged image of the circular area in the left image: Turning-RNBE, such as RNBE1 and RNBE3, Straight-RNBE, such as RNBE2, and particle derivation locations, such as the red triangle and red dots.

Figure 5a shows the road network map, and the enlarged image of the intersection in the red circle is shown in Figure 5b, where the red arrow indicates the heading direction of the vehicle. We mark the RNBE to which the vehicle currently belongs as RNBEcur. When RNBEcur changes, the planned-driving RNBEs are obtained based on the connectivity between RNBEs calculated in the initial stage. There are three planned-driving RNBEs for the vehicle at the road intersection in Figure 5b, which are marked RNBE1, RNBE2, and RNBE3, separately. We calculate the angle φ between the Head-Orientation of each planned-driving RNBE and the Tail-Orientation of RNBEcur. If φ is greater than 40°, we mark the planned-driving RNBE as Turning-RNBE, such as for RNBE1 and RNBE3, and if φ is less than 40° we mark the planned-driving RNBE as Straight-RNBE, such as for RNBE2.

The selected map correction points include Turning-Tp correction points and Straight-Tp correction points, which correspond to Turning-RNBE and Straight-RNBE, respectively. 

When the planned-driving RNBE is Turning-RNBE and unique, we calculate the angle φ between the Head-Orientation of the Turning-RNBE and the Tail-Orientation of RNBEcur. At the same time, we calculate the angle $\beta_{1}$ between the vehicle heading and the Tail-Orientation of RNBEcur, and the angle $\beta_{2}$ between the vehicle heading and the Head-Orientation of the Turning-RNBE. If the above variables satisfy Equation (6), the vehicle meets the selecting condition for the Turning-Tp correction point.

(6) $$\left(\beta_{1}>0.6\varphi \right)\&\&\left(\beta_{2}<0.4\varphi \right)$$

The vehicle can only meet the above conditions after completing most of the steering actions. We translate the Tp by half the width of the road along the heading of the Head-Orientation of the Turning-RNBE, as shown in the red dots mark in Figure 5b, and then derive particles from it. Finally, we use the Head-Tp of RNBEcur as the reference point and apply the similarity judgment criterion to obtain the most similar particle, which is the Turning-Tp correction point.

When the planned-driving RNBE is Straight-RNBE and unique, we mainly set selecting criteria based on the distances between the vehicle position and Nodes in the map. For the convenience of introduction, we use $HTp_{Cur}$ as the Head-Tp of RNBEcur, and $P_{gi}$ as the vehicle position at the i moment. We calculate the distance $Len_{VO\_Last}$ between $HTp_{Cur}$ and $P_{gi-1}$, and the distance $Len_{VO\_Cur}$ between $HTp_{Cur}$ and $P_{gi}$, and we predict the distance $Len_{VO\_Next}$ between $HTp_{Cur}$ and $P_{gi+1}$ by assuming that the vehicle travels at a constant speed. The distance between the Head-Tp and Tail-Tp of RNBEcur is $Len_{TPs}$. If the above variables satisfy Equation (7), it means that the vehicle is passing through the road intersection for the first time. Another condition is if the variables satisfies Equation (8), which means that the vehicle has not passed the road intersection at the current moment, but the vehicle will pass the road intersection at the next moment and be closer to Tp than this moment. It should be noted that since the original map only contains the longitude and latitude information of Nodes, the distance calculations mentioned above only consider the position difference in longitude and latitude directions.

(7) $$\left(Len_{VO\_Last}<Len_{TPs}\right)\&\&\left(Len_{VO\_Cur}>Len_{TPs}\right)$$

(8) $$\left(Len_{VO\_Cur}<Len_{TPs}\right)\&\&\left(Len_{VO\_Next}>Len_{TPs}\right)\&\&\left(\left|Len_{VO\_Cur}-Len_{TPs}\right|>\left|Len_{VO\_Next}-Len_{TPs}\right|\right)$$

The above conditions can be met when the vehicle reaches the intersection of the roads. We derive particles from the Tail-Tp of RNBEcur, as shown in Figure 5b, marked with the red triangle. Finally, we use the Head-Tp of RNBEcur as the reference point and apply the similarity judgment criterion to obtain the most similar particle, which is the Straight-Tp correction point.

When the planned-driving RNBE is not unique, if the vehicle meets the Turning-Tp selecting criteria, we change the RNBE to which the vehicle belongs to the corresponding Turning-RNBE. When the vehicle meets the Straight-Tp selecting criteria, we still preserve the possibility of changing the RNBE to which the vehicle belongs because of the cumulative drift error. Until the distance between the vehicle and the Head-Tp of Straight-RNBE reaches more than 10 m, we change the belonging RNBE to Straight-RNBE. When the belonging RNBE changes, we adjust the Head-Tp, Tail-Tp, and internal Sps′ order of the planned-driving RNBEs.

##### Sp Correction Point Selection

For the Sp correction points, we adopt the judgment method similar to Straight-Tp correction points, where $Len_{VO\_Last}$, $Len_{VO\_Cur}$, and $Len_{VO\_Next}$ have the same calculation method, as mentioned earlier. The distance between Sp and the Head-Tp of RNBEcur is $Len_{SP-TP}$. If the above variables satisfy Equation (9) or Equation (10), the location of the vehicle has met the selecting condition for the Sp correction point.

(9) $$\left(Len_{VO\_Last}<Len_{SP-TP}\right)\&\&\left(Len_{VO\_Cur}>Len_{SP-TP}\right)$$

(10) $$\left(Len_{VO\_Cur}<Len_{SP-TP}\right)\&\&\left(Len_{VO\_Next}>Len_{SP-TP}\right)\&\&\left(\left|Len_{VO\_Cur}-Len_{SP-TP}\right|>\left|Len_{VO\_Next}-Len_{SP-TP}\right|\right)$$

To avoid the impact of Straight-Tp correction points, we start with reverse order selecting from the end of the Sp group. The particles are derived from Sps that meet the selecting criteria, and we directly skip those Sps that do not meet the conditions. Finally, we, respectively, use the Head-Tp and Tail-Tp of RNBEcur as the reference points and apply the similarity judgment criterion to obtain the most similar particle, which is the Sp correction point.

### 4.3. Trajectory Optimization

**Figure 6:**
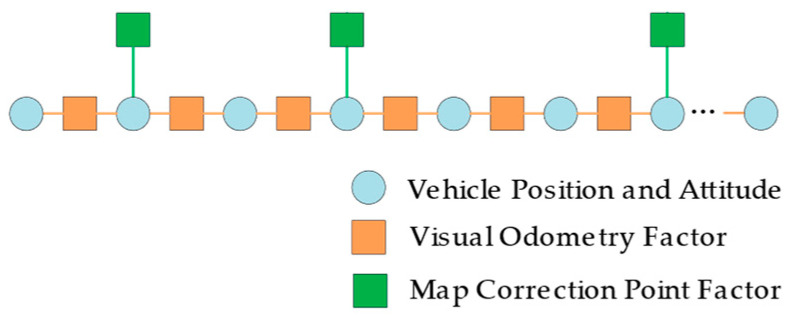
An illustration of the global pose graph structure. Every node represents one position and attitude of the vehicle in the ENU coordinate system. The edges between consecutive nodes are local constraints from VO. Other edges are global constraints from map correction points.

This section optimizes the trajectory by simultaneously considering the short-term accuracy of the original output of VO and the global consistency of the map correction points. We constructed the global pose graph, as shown in Figure 6. The nodes in the pose graph represent the position and attitude of the vehicle at different times. The VO system provides short-term constraints for consecutive nodes, and the Tp/Sp correction points provide global constraints for uncertain intervals. The pose graph optimization problem is essentially a maximum likelihood estimation problem. The maximum likelihood estimation consists of the joint probability distribution of the position and attitude of the vehicle in the ENU coordinate system over a period of time.

We assume that the output of VO is short-term accurate, which means that the relative errors between adjacent frames are small. This assumption applies to the vast majority of existing VO methods. We assume that $T_{ci}^{w}$ and $T_{cj}^{w}$ are the outputs of VO at i and j moments, both of which are invertible. Then, we convert them to the ENU coordinate to obtain $T_{ci}^{enu}=T_{ci}^{w}T_{w}^{enu}$ and $T_{cj}^{enu}=T_{cj}^{w}T_{w}^{enu}$. For the convenience of explanation, we use $T_{i}^{enu}$ and $T_{j}^{enu}$ to represent the conversion of the VO outputs at i and j moments, and use $T_{i}^{j}={\left(T_{j}^{enu}\right)}^{-1}T_{i}^{enu}$ to represent the conversion relationship between adjacent frames, the Lie algebraic form of which is $\varepsilon_{ij}=In{\left[{\left(T_{j}^{enu}\right)}^{-1}T_{i}^{enu}\right]}^{\vee }$. Due to the reversibility of $T_{ci}^{w}$, $T_{cj}^{w}$, and $T_{w}^{enu}$, $T_{i}^{enu}$ and $T_{j}^{enu}$ are always invertible and the above calculations can always be made.

We add left perturbations $\delta \varepsilon_{i}$ and $\delta \varepsilon_{j}$ to $\varepsilon_{i}$ and $\varepsilon_{j}$, as shown in Equation (11), where $\varepsilon_{i}$ and $\varepsilon_{j}$ are the Lie Algebraic forms of $T_{i}^{enu}$ and $T_{j}^{enu}$, and $\overset{\wedge }{\varepsilon_{ij}}$ represents the conversion term after adding perturbations.

(11) $$\begin{array}{ll} \overset{\wedge }{\varepsilon_{ij}} & =In{\left[{\left(T_{j}^{enu}\right)}^{-1}exp\left({\left(-\delta \varepsilon_{j}\right)}^{\wedge }\right)exp\left({\left(\delta \varepsilon_{i}\right)}^{\wedge }\right)T_{i}^{enu}\right]}^{\vee }\\ & =In{\left[{\left(T_{j}^{enu}\right)}^{-1}T_{i}^{enu}exp\left({\left(-Ad\left({\left(T_{i}^{enu}\right)}^{-1}\right)\delta \varepsilon_{j}\right)}^{\wedge }\right)exp\left({\left(Ad\left({\left(T_{i}^{enu}\right)}^{-1}\right)\delta \varepsilon_{i}\right)}^{\wedge }\right)\right]}^{\vee }\\ & \approx In{\left[{\left(T_{j}^{enu}\right)}^{-1}T_{i}^{enu}\left(I-{\left(Ad\left({\left(T_{i}^{enu}\right)}^{-1}\right)\delta \varepsilon_{j}\right)}^{\wedge }+{\left(Ad\left({\left(T_{i}^{enu}\right)}^{-1}\right)\delta \varepsilon_{i}\right)}^{\wedge }\right)\right]}^{\vee }\\ & \approx \varepsilon_{ij}-J_{r}^{-1}\left(\varepsilon_{ij}\right)Ad\left({\left(T_{i}^{enu}\right)}^{-1}\right)\delta \varepsilon_{j}+J_{r}^{-1}\left(\varepsilon_{ij}\right)Ad\left({\left(T_{i}^{enu}\right)}^{-1}\right)\delta \varepsilon_{i} \end{array}$$

The corresponding Jacobian matrixes are shown in Equations (12) and (13).

(12) $$\frac{\partial \varepsilon_{ij}}{\partial \delta \varepsilon_{j}}=-J_{r}^{-1}\left(\varepsilon_{ij}\right)Ad\left({\left(T_{i}^{enu}\right)}^{-1}\right)$$

(13) $$\frac{\partial \varepsilon_{ij}}{\partial \delta \varepsilon_{i}}=J_{r}^{-1}\left(\varepsilon_{ij}\right)Ad\left({\left(T_{i}^{enu}\right)}^{-1}\right)$$

where $J_{r}^{-1}\left(\varepsilon_{ij}\right)\approx I+\frac{1}{2}\left[\begin{array}{cc} \phi_{\varepsilon_{ij}}^{\wedge } & \rho_{\varepsilon_{ij}}^{\wedge }\\ 0 & \phi_{\varepsilon_{ij}}^{\wedge } \end{array}\right]$ and Ad(Tx)=[Rxtx^Rx0Rx]. $\phi_{\varepsilon_{ij}}$($R_{x}$) and $\rho_{\varepsilon_{ij}}$($t_{x}$) represent the rotational component and translation component of $\varepsilon_{ij}$($T_{x}$), respectively.

The Tp/Sp correction points selected in Section 4.2 only contain longitude and latitude information. We assign the height information of the vehicle position that meets the selecting criteria to correction points. Then, we calculate the coordinate of the correction point in the ENU coordinate system as the map correction factor and add it to the pose graph. After constructing the global pose graph, the optimization process is equivalent to searching for the configuration of nodes that matches all edges as much as possible. We design a time-varying sliding window for pose graph optimization to obtain accurate and globally drift-free estimation, which can save computing power effectively.

**Figure 7:**
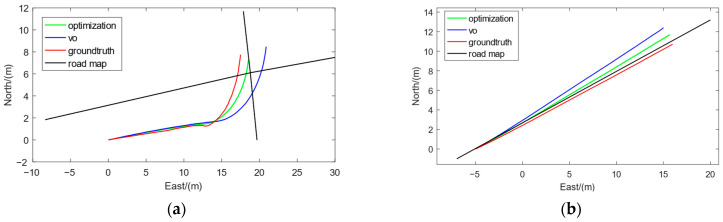
The black lines represent the road network map, the blue line represents the original trajectory, the green line represents the optimized trajectory, and the red line represents the ground truth. (**a**) Correction effect of Turning-Tp correction points. (**b**) Correction effect of Straight-Tp/Sp correction points.

The selection of Turning-Tp correction points mainly depends on the angle judgment criterion and has high stability. As shown in Figure 7a, we use Turning-Tp correction points to correct the trajectory length. The calculation window for the Turning-Tp designed in this paper starts from the previous Turning-Tp correction point. In particular, the initial vehicle position is used as the first Turning-Tp correction point in the initial stage.

The selection of Straight-Tp/Sp correction points mainly depends on the length judgment criterion, which is mainly used to correct the direction of the original trajectory, as shown in Figure 7b. Due to the inherent limitations of the dead-reckoning method, a little heading deviation may lead to significant positioning errors as the distance traveled increases. We design the calculation window for the Straight-Tp/Sp correction points to start from the first 1000 frames of the current time. If the number of Turning-Tp points within the first 1000 frames is less than five, the range of the calculation window will be expanded to 1500 frames. If the existing frame count is less than 1000/1500, we use all frames to optimate.

## 5. Experimental Results

**Figure 8:**
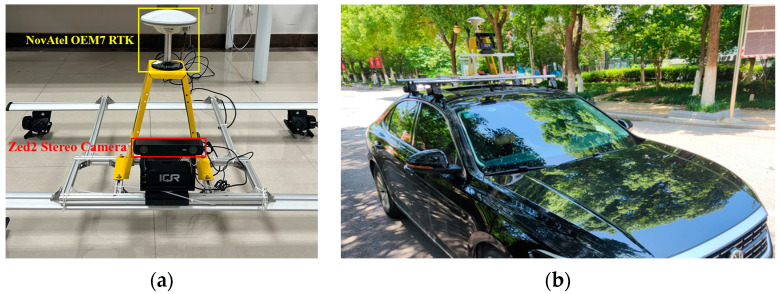
Image (**a**) shows the onboard experimental platform, with the ZED2 stereo camera in the red box and the RTK device in the yellow box, and (**b**) shows the actual installation effect of the vehicle-mounted experimental platform.

To evaluate the algorithm proposed in this paper in real situations, we conducted experiments on the campus dataset and the KITTI dataset [49]. We collected visual images with the ZED2 stereo camera and obtained the truth trajectory using the device of NovAtel OEM7 RTK in the campus experiment. We used the vehicle to collect the campus dataset, as shown in Figure 8.

In all experiments, we used the VO part of ORB_SLAM2 [18] as the original input. We set the weight α for Turning-Tp correction points to 0.7, and the weight α for Straight-Tp/Sp correction points to 0.6. The number of derived particles for each node was set to 300. We only calculated the positioning errors in the longitude and latitude direction to evaluate the trajectory accuracy.

### 5.1. Comparison with a Traditional VO Algorithm

**Figure 9:**
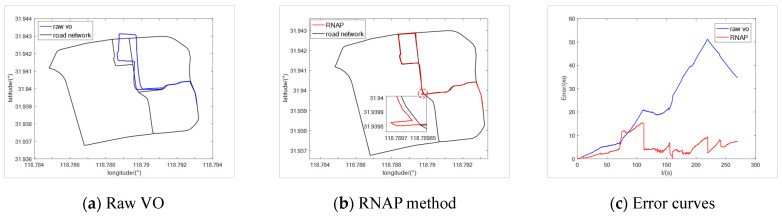
Experimental results of the campus dataset.

Firstly, we compared the proposed algorithm with the original VO algorithm on the campus dataset. The trajectory of VO and the regional road network map are shown in Figure 9a, where the positioning error of VO rapidly increases over time, and the vehicle trajectory gradually deviates from the road range. After adding the algorithm proposed in this paper, the positioning error of VO was suppressed effectively, as shown in Figure 9b. We enlarged the red circular area, as shown in the small figure of Figure 9b, and the displacement error of the vehicle could not be corrected within the RNBE, and the positioning error gradually increased like the original trajectory. But after detecting the Turning-Tp correction point, the displacement error correction was quickly completed. And the heading correction was completed through the internal Sp correction points of the next RNBE. The time-varying curve of vehicle positioning error is shown in Figure 9c, from which we can see that the method proposed in this paper can introduce constraints from the point-line form road network map, which can suppress positioning error. After selecting the map correction points of the road network, the method proposed in this paper can fully utilize their information and quickly improve vehicle positioning accuracy. Compared to the original trajectory, the average positioning error decreased from 23.206 m to 5.179 m.

**Figure 10:**
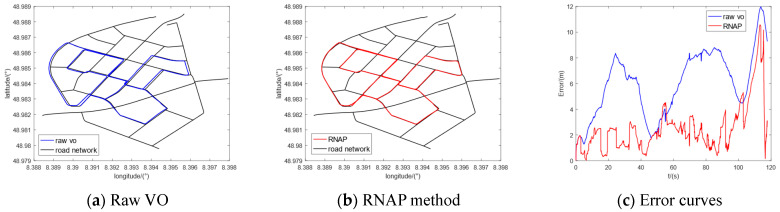
Experimental results of KITTI-00.

**Figure 11:**
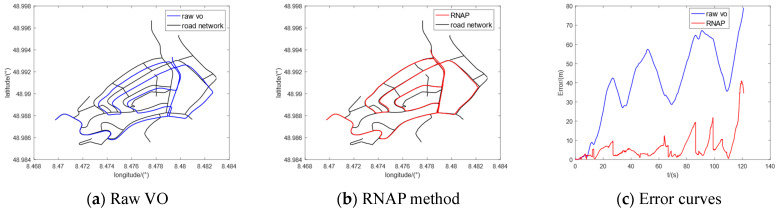
Experimental results of KITTI-02.

**Figure 12:**
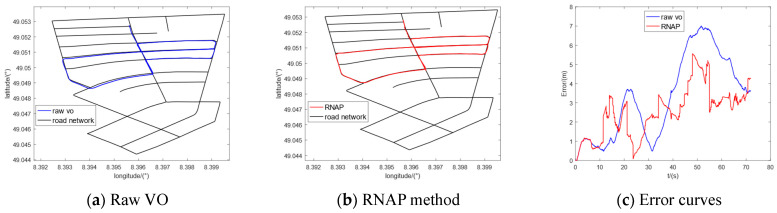
Experimental results of KITTI-05.

**Figure 13:**
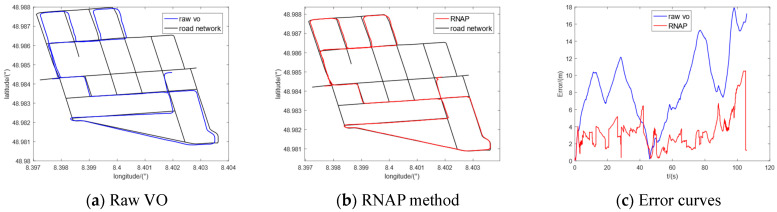
Experimental results of KITTI-08.

**Figure 14:**
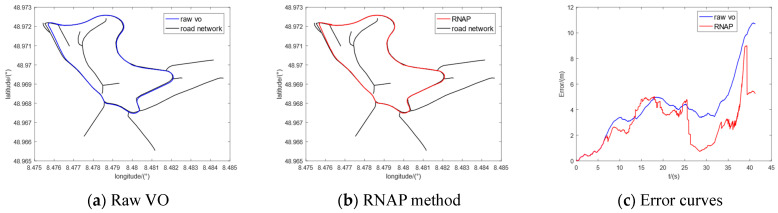
Experimental results of KITTI-09.

Furthermore, we selected five sequences (00, 02, 05, 08, and 09) in the KITTI dataset to conduct the comparative experiments. The regional maps where they are located have multiple intersections or high-curvature roads, which contain enough map information to correct the original trajectory of VO. The experimental results are shown in Figure 10, Figure 11, Figure 12, Figure 13 and Figure 14, from which we can see that the algorithm proposed in this paper has good trajectory correction performance and is suitable for curves and linear trajectories within relevant sequences. The (c) subfigures in Figure 10, Figure 11, Figure 12, Figure 13 and Figure 14 show a comparison of the positioning error over time when applying the original VO and the method proposed in this paper. As the driving time increased, the positioning error of the original VO system gradually increased. Our method can effectively suppress the positioning error and reduce the average positioning error from 12.47 m to 3.50 m.

### 5.2. Comparison with Other Localization Algorithms

We compared the method proposed in this paper with other map-based algorithms, of which the closest ones to our method were [42,45,46,47,48], all of which introduce constraints to correct trajectories from the point-line form map. The Snap method [42] divides the original trajectory into multiple straight-line segments, then matches the set of line segments with given maps composed of line segments to retrieve the most similar route among them. The Graph-Based method [45] does not require external sensors. This method extracts a sequence of heading length values through autonomous sensors and matches them with a preprocessed heading length map for localization. The Openstreetslam [46] method is based on trajectory-fast chamfer matching technology and uses a particle filter to correct the position of the trajectory at each cycle. The TP Filter method [47] collects turning points of the trajectory and uses a particle filter to match the turning points with the turning points of the regional map. The TP Filter method can effectively suppress positioning errors, but it is not suitable for high-curvature road sections. The MPJPF method [48] puts forward the concept of the anchor point and its selecting method. Like [47], MPJPF uses a particle filter to match the anchor points with the anchor points of the regional map to suppress the positioning error. Compared with [47], it has a strong application ability for high-curvature road sections.

The comparison results between the method proposed in this paper and the other five methods are shown in Table 1, where A_E represents the Average-Error and M_E represents the Max-Error. Our method introduced constraints from the point-line form road network map, which had higher positioning accuracy than the above five methods. Compared to the MPJPF method with the strongest applicability and highest positioning accuracy, the average positioning error was reduced by 23.49%. In the KITTI-02 sequence, our method performed poorly in terms of the max error. The main reason is that the height of the vehicle driving trajectory in the 02 sequence varies greatly. But there was no height information constraint in the original map. In the future, the introduction of 3D maps for improvement can be considered.

The Snap [42] and TP Filter [47] methods suppress drift errors by selecting turning points of the trajectory. They can only be applied to sequences like 05 and 08, whose roads are almost straight, and they failed in high-curvature road sections in sequences 00, 02, and 09. This is because their correction point selecting methods are overreliant on turning points that are not present on curve roads. Compared to the Snap and TP Filter methods, the method proposed in this paper reduces the average error by 27.78% and 24.61%, respectively. The OpenStreetslam method [46] has a lower average positioning accuracy, partly due to the relatively low accuracy of the original trajectory. The main reason is that this method has insufficient robustness to the distortion of the output of VO, which can easily lead to incorrect matching and reduced positioning accuracy. The Graph-Based method [45] has been tested on the 00 and 05 sequences and does not rely on external information. The assistance provided by the sequence of heading length values in 02 and 09 with significant altitude changes may be limited. The method proposed in this paper can achieve better positioning accuracy than the method above. The average positioning error in the 00 and 05 sequences decreased by 31.5%. The MPJPF method [48] uses multi-position joint filtering for matching, which can reduce the risk of mismatching. But the severity of the consequences of mismatching is also increased. At the same time, the positioning accuracy that can be improved is limited in sequences 05 and 09. Correction points selected by the method proposed in this paper are independent. The impact of a single mismatch will soon be compensated for by subsequent correction points. At the same time, the above methods rely on segmenting and matching existing trajectories. The corrected positions of each frame are calculated through a simple proportional relationship after matching, which has hysteresis. However, the method proposed in this paper can provide a real-time optimal estimation of vehicle position by the optimization and prediction model.

The experiment in this paper was conducted on a computer equipped with an Intel i7 2.3 GHz CPU and 16 GB of memory. The average selecting time for each Turning-Tp correction point was 0.143 ms, and the average selecting time for each Straight-Tp/Sp correction point was 0.227 ms. The average optimization time for each Turning-Tp correction point was 5.795 ms, and the average optimization time for each Straight-Tp/Sp correction point was 33.432 ms. The sampling period of the camera in both the campus dataset and the KITTI dataset was 200 ms. The average single-frame image processing time of VO was 49.21 ms. We adopted the map correction point selecting and optimization mechanism, where only sub-millisecond level selecting was added during each single-frame image processing. The relatively time-consuming optimization stage was only enabled when the image frame meets the selecting conditions, which accounted for 2.8% to 16.7% of the overall cycle. The method proposed in this paper added minimal time consumption to the traditional VO, which can effectively improve the vehicle positioning effect and is very cost-effective.

## 6. Conclusions

Aiming at the problem of cumulative drift error in VO, this paper proposes a road-network-assisted vehicle positioning method based on the theory of pose graph optimization. To simplify and efficiently utilize road network map information, we introduced a new road network model based on the concept of RNBE. Then, we proposed the RNAP algorithm, which uses real-time prediction results of vehicle positioning and road network map data to select various correction points. The algorithm constructs a global pose graph to optimize the original trajectory and predict the latest position and attitude of the vehicle. Our method only relies on the original output of VO and the regional road network map, requiring low storage space and computational cost. The experimental results on both campus and public datasets show that the proposed method can effectively reduce vehicle positioning errors, providing a new method for long-term vehicle positioning that does not rely on satellites or other types of absolute position sensors.

## Figures and Tables

**Table 1:** Positioning errors for different methods.

Method	00		02		05		08		09	
	A_E	M_E	A_E	M_E	A_E	M_E	A_E	M_E	A_E	M_E
VO	6.02	12.01	40.46	79.30	3.35	7.24	8.49	17.58	4.05	10.82
Snap [42]	Failed	Failed	Failed	Failed	4.0	8.6	4.1	15.8	Failed	Failed
Graph-Based [45]	~2.8	~7.7	—		~4.5	~8.0	—		—	
OpenStreet-SLAM [46]	~11.5	~28	~22.3	~55	—		—		—	
TP-Filter [47]	—		—		3.92	8.15	3.84	14.62	—	
MPJPF [48]	3.5	9.2	7.6	20.4	3.3	7.4	3.9	11.1	4.6	10.6
RNAP	2.48	10.57	6.32	41.05	2.52	5.56	3.33	10.55	2.87	8.99

## Data Availability

Not applicable.
